# Emissions Production by Exhaust Gases of a Road Vehicle’s Starting Depending on a Road Gradient

**DOI:** 10.3390/s22249896

**Published:** 2022-12-15

**Authors:** Branislav Šarkan, Michal Loman, František Synák, Tomáš Skrúcaný, Jiří Hanzl

**Affiliations:** 1 Faculty of Operation and Economic of Transport and Communications, University of Zilina, Univerzitná 8215/1, 010 26 Žilina, Slovakia; 2 Faculty of Technology, Institute of Technology and Business in České Budějovice, Okružní 517/10, 370 01 České Budějovice, Czech Republic

**Keywords:** emissions, vehicle, driving tests, road gradient, environment, air quality

## Abstract

An increasing number of motor vehicles are connected with negative environmental impacts in relation to their operation. Among the main negative effects are exhaust gas emissions production. The annual increase in passenger cars and emissions from them deteriorates air quality daily. Traffic junctions also have a negative impact on increasing emissions production by exhaust gases. This situation may be caused by vehicle speed fluctuation, mainly when they get closer or leave. This study focuses on the emissions produced by exhaust gases after a road vehicle starts. The research was performed with a combustion engine vehicle on a route 30 m long. The vehicle was simulated in three different ways of starting (uphill, on ground level/plain and downhill). The values of carbon monoxide (CO), carbon dioxide (CO_2_), hydrocarbons (HC) and nitrogen oxides (NO_X_) were observed, as well as the vehicle’s operation performance during start-ups. The research results showed that the lowest emissions production is when the vehicle is starting downhill. There, the emissions increased up to a distance of 9.7 m from the start. After reaching this distance, the emissions decreased and the vehicle speed continued to increase. While the vehicle started uphill, the emissions increased up to the distance of 16.8 m. After reaching this distance, the emissions began decreasing. Due to this fact, this type of testing is assessed as “the worst” from the emissions production point of view. The research demonstrates the relations between a road gradient representing starting on a plain surface and a vehicle’s emissions produced by the exhaust gases. It is known that exhaust emissions are higher predominantly at junctions. They depend considerably on vehicle speed and driving continuity on a route. This research helps to quantify all the data and, thus, to provide a possibility of further solutions in the future as a tool for emissions reduction in cities and close to traffic intersections.

## 1. Introduction

Road traffic can be classified as a significant sector in terms of energy consumption and production of emissions. Such productions are rising due to economic and population growth [1,2]. International Energy Agency (IAE) data from 2017 show that energy consumption in traffic increased from 23% for overall final consumption of the year 1971 to 29% in the year 2015 [3]. Meanwhile, air quality is getting poorer, which continues to be a critical public health issue around the world. The World Health Organisation (WHO) estimates that ambient air pollution causes 4.2 million premature deaths per year worldwide [4]. Although several things are contributing to this problem, road traffic is still the main source of air pollution in city areas [5,6,7]. Passenger cars are one of the greatest CO_2_ emitters, and they even have a 60.6 percent share of the total emissions produced in traffic. Regarding the average number of passengers in a single car, which was 1.6 people in 2018, it is possible to reduce the emissions alternatively by sharing the cars, using public transportation or bicycles, and walking [8,9]. Currently, there is a big effort to reduce energy consumption and emissions of pollutants from motor vehicles. In connection with this issue, safety in road transport is also increasing [10]. According to Rezaei et al. [11], a hybridisation of vehicles’ driving system is one of the technologies to solve these problems.

Concerning the research, it is necessary to pay attention mainly to the places where emissions affect people directly. These places can be the built environment. The research authors observed the impact of a vehicle starting on the emissions production. Prior to the research itself, one must be aware that the seriousness of the problem increases just when the traffic flow is interrupted. Then, it leads to delays and stopping. Such situations regularly happen at traffic junctions and places where the traffic intensity is so high that the communication capacity is not sufficient. This phenomenon often makes vehicles stop and start again. The characteristics related to traffic in combination with characteristics of a road and vehicle increase emissions at traffic junctions and places where rows of vehicles occur.

The level of emissions production depends on several factors, especially traffic characteristics, vehicle type and the organization of junctions or roads [12,13]. For instance, the type of a vehicle, its weight and age, the engine condition and performance, as well as their maintenance, all relate to the amount of emissions produced by the given vehicle [14,15,16]. In addition, the fuel quality directly affects the exhaust gas emissions [17,18].

Road transport is still the major contributor to air pollution, especially in urban areas with high traffic intensity. Many researchers estimate that the air pollution in Europe causes almost 500,000 premature deaths yearly [19]. The most critical pollutants are nitrogen oxides (NO_X_) and particulate matter (PM_2,5_) that are emitted mainly by vehicles with a compression ignition engine. To improve the air quality sustainably, European legislation has set stricter limits. From September 2017, within the framework of type approval, new tests called RDE—real drive emission—have been performed. Driving tests have been established in order to measure the emissions of all new car models during real driving conditions [19,20,21,22]. According to the latest available IEA report from 2020, the transport sector accounts for up to 32% of energy consumption. In 2020 and 2021, there was a slight decrease in energy consumption in the transport sector (8–14%) due to the COVID-19 pandemic. While electrification is gathering pace, oil products still dominate the sector, providing around 91% of its final energy use. Road transport makes up around 75% of energy demand and emissions [23].

In recent times, many researchers have paid attention to measuring real emissions while vehicle driving. The results have shown that real emissions often differ significantly from the emissions measured under laboratory conditions by standard procedures [24,25].

Laboratory testing does not take into consideration many factors that can have an impact on real driving emissions production [26]. The research results with compression ignition engine vehicles show that temperatures under 20 °C in some vehicles can cause an insufficient system processing of NO_X_ emissions reduction. In this case, it may be due to the catalytic converters and EGR valves. Sometimes it even leads to total dysfunction of these systems. It is interesting that in relation to type approval, the ambient temperature is prescribed as more than 20 °C. Therefore, the system’s malfunction or dysfunction cannot be found [27,28]. The results of other research point to an increase in emissions production during type approval testing at low temperatures. In these cases, NOx emissions increase threefold. Similar results have been observed in diesel and spark-ignition engines [29]. Interestingly, the same results are also determined at high temperatures (above 30 °C). If a driving cycle of type approval Euro 6 is performed at a low temperature of −7 °C, NOx emissions increase more than threefold in the vehicles with spark-ignition, as well as diesel engines [30]. The same malfunctions were found in testing with very high ambient temperatures (above 30 °C) or when starting up a warm engine (i.e., oil temperature higher than 80 °C) [31,32].

The objective of this study is to analyse the impact of road gradient on exhaust emissions. The measurements were performed with a combustion engine vehicle. The exhaust emissions were studied while the vehicle was starting on a 30 m route. During the driving tests, CO, CO_2_, HC and NOx emissions were observed by a MAHA MGT5 analyser of the exhaust gases. The vehicle drove on an asphalt surface on a route with different height profiles. The vehicle emissions were observed at positive route slope (uphill), negative route slope (downhill) and level ground (plain).

The overall analysis of the measurement results were focused on the impact of the road gradient on the amount of selected components of the exhaust gases produced. To this purpose, it is discussed whether the road gradient really affects the exhaust emissions production and if so, to what extent. This finding represents the main part of the research. At present, there are different programs used to model traffic situations. However, the microscopic models used most often for lightweight vehicles do not consider the road gradient in the total calculation of the emissions load. The study of DW Wyatt et al. [33] identified the impact that the road gradient can have on modelling CO_2_ emissions on a micro level and developed new methodology to include the road gradient in modelling. Likewise, it is necessary to take into consideration the junctions’ slopes. When designing models, they must pay attention to emissions production while the vehicle is starting [34].

## 2. Experimental Activity

There is a need to maintain the similarity of individual cycles during the driving tests. For that reason, a vehicle with automatic gear was selected for the measurements. When testing, the vehicle measured simulates a standard start at the junction. After moving the vehicle off, its speed increases fluently. The measurement were stopped after driving a route 30 metres long. All the time during vehicle starting, the data from the engine control unit and the amount of exhaust gas emissions production were recorded. To observe the amount of emissions, the vehicle drove on the asphalt route with a different gradient. Firstly, the vehicle started on level ground. Secondly, the vehicle started uphill. Finally, the vehicle started downhill. The measurements were repeated 20 times for all types of starting. The courses of each starting were almost identical. After driving the route measured, the measurement was finished and the data were saved. Starts with extreme values were excluded from further research. All the measurements were performed on the same principle. There were 60 measurements together (20 measurements for each route gradient) performed for the study objectives.

The whole process and calculation of the amount of emissions produced is managed under Regulation 2017/1151. Based on the calculation of emissions according to this regulation, it is possible to determine the emissions of specific components in g/s. Particular processes to determine the amount of exhaust emissions are also given in respected publications [35,36]. Thus, the summarization of the points provides the weight of specific emissions components for the entire duration of the vehicle starting. In order to further process the data to a comparable value (g/s), it is necessary to make several calculations. An important part of the measurement is data from the engine control unit, which are recorded by diagnostics via OBD connection. For the calculation of specific emissions components, the following data were recorded:

- RPM;
- Vehicle speed;
- Mass air flow.

The data were recorded in two different ways:

- Exhaust emission production—analyser of the exhaust gases;
- Data from the engine control unit—diagnostic device Vgate Icar OBD.

After data exporting to the PC, a mutual synchronization was needed. For simplicity, the record frequency was set at 0.5 s to ensure the possibility of exported data matching. The detailed description of the methodology of calculating the specific exhaust gas components are elaborated by Kuranc et al. [37].

### 2.1. Vehicle Description

**Figure 1:**
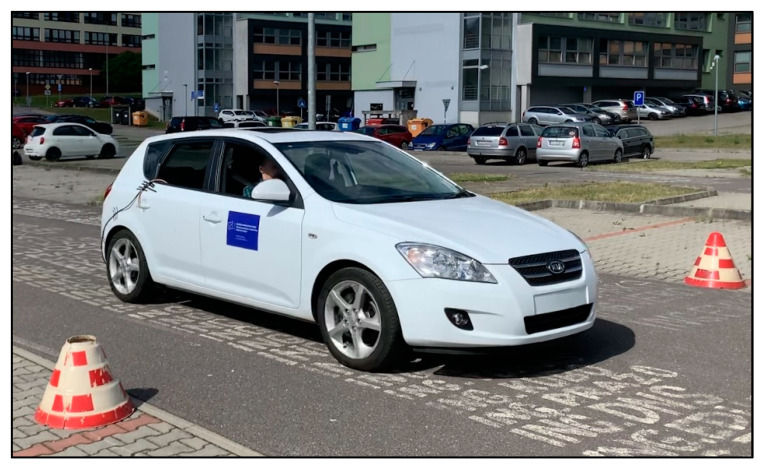
Measuring vehicle [author].

Driving tests were performed with the Kia Ceed (Figure 1). Prior to the driving tests, the vehicle was checked. It was important to ensure that the research results would not be affected by hidden vehicle malfunctions. For this reason, the vehicle underwent a static emissions inspection in so far as the regular emissions inspection. In the Slovak Republic, an emissions inspection is obligatory for passenger cars every two years. In order to exclude any malfunction of the engine management, another check was performed by OBD diagnostics using BOSCH KTS 560. The engine control unit had no error codes saved in the memory of defects.

The technical data of the vehicle measured are given in Table 1. The actual vehicle mass differs from the mass given in the table. As follows from [38], the amount of emissions production depends on the actual vehicle mass. Due to this fact, the vehicle was weighed by the mobile weighing machine Tenzo PW10 prior to the measurements. When weighing, the vehicle was loaded with all the devices needed for measuring, and there were also two people in the vehicle who operated the measuring equipment and were driving. The resulting vehicle mass was 1315 kg at the driving tests.

From the comparison of the weights, it can be observed that the vehicle was loaded during the measurement in compliance with the values given by the manufacturer. When comparing with the maximum permissible total weight, it is possible to conclude that the vehicle was employed by 73% during the measurement. The weight check was performed after measuring as well. The portable scale did not detect any change in weight. In fact, the only change was caused by the reduction of fuel in a tank during the measurement. This change in weight had a negligible impact on the emissions production.

### 2.2. Measuring Devices

The values of the exhaust emissions were monitored during the driving tests. To collect the data on emissions production continually, the MAHA MGT 5 exhaust gas analyser was placed in the vehicle. Data collection was done by an exhaust gas probe that was located in the exhaust pipe’s opening. The analyser’s charging was provided through an external battery. According to [39], each device that is connected externally increases fuel consumption and, thus, the emissions production to a certain extent. Information on the combustion engine’s functioning was recorded by an OBD connection continually. To record the data, the Vgate Icar OBD and the OBD Fusion mobile application were used. The data recording frequency of the measuring devices was 0.5 s. The technical data of the exhaust gas analyser are given in Table 2.

**Figure 2:**
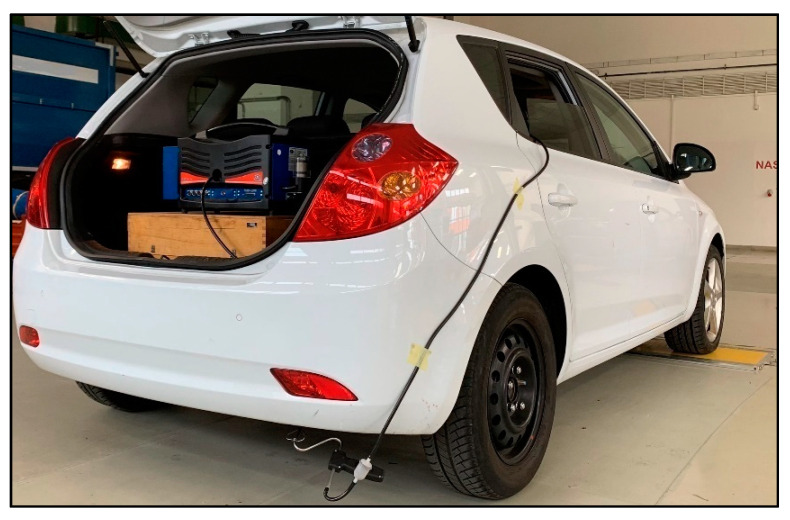
Measuring vehicle before measurement [author].

The analyser was able to detect the following values of emissions in the exhaust pipe: HC, CO, CO_2_ and O_2_, with a calculation of lambda. To improve the research, the analyser was also accompanied by a NOx sensor, which is an uncommon part of the device. The analyser was primarily designed for measuring the emissions of a standing vehicle (e.g., during the emissions inspection). The device is regularly calibrated in accordance with the national law of metrology. The data are processed by specialized software—Maha Emission Viewer. It enables continuous recording of the exhaust gas components (in % or ppm). The vehicle together with the measuring equipment is shown in Figure 2. The picture was taken shortly before the measurement realization.

The MAHA MGT 5 analyser of the exhaust gases can be seen in the luggage compartment. Connection of the exhaust gas probe to the analyser was done through the rear window, which was open during the measurement. The window was open only to the width needed for the hose, which ensured the gas intake from the exhaust to the body of the analyser. In terms of low speeds during the starts, there was no assumption of the results being distorted. The impact of air resistance on fuel consumption is considerable at higher speeds, as seen in [41].

### 2.3. Measuring Route

Three types of measuring routes were chosen for this study. Technical information on the measuring routes is given in Table 3.

In this study, emphasis was placed on the correct selection of a measuring route. From Figure 1, it can be seen that each measuring section has an adequate length for measuring and a safety stop after driving the section. The wind speed check was done prior to the measurement. Various studies [42,43] mention the impact of wind speed on fuel consumption. During the measurement, the wind speed measured was only 20 m·s^−1^. Such low wind speed is negligible in terms of the driving tests.

The altitude value of the measuring section is 378 m above sea level. This value is general for the entire territory of the city where the tests were conducted. The ambient air temperature was 25 °C

**Figure 3:**
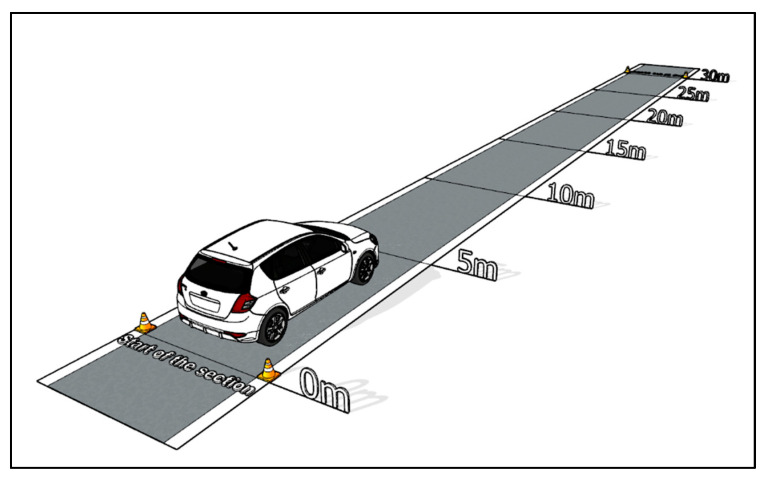
Measurement Visualization—Plane [author via Sketchup].

The first type of start-up test was performed on the route with a slope of 0° (plain). A driving test simulation is shown in Figure 3. The measurement started after the measuring equipment was applied. Then, a flow vehicle’s starting began. All start-ups represent a standard vehicle start from the speed of km·h^−1^. The measurement was finished after driving the route 30 m long. The measuring equipment was turned off after the vehicle stopped. All 20 measurements were performed in the same way. The assessment of the start-up tests was limited to the time when the driver applied the gas pedal until the time when the vehicle drove 30 m.

**Figure 4:**
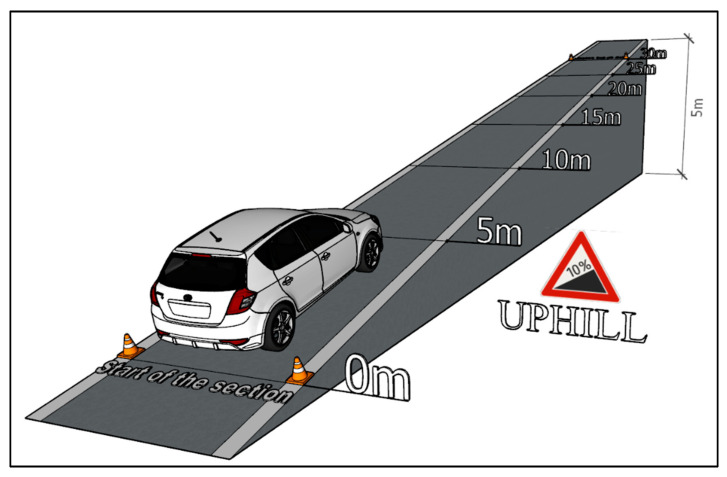
Measurement Visualization—Uphill [author via Sketchup].

The second type of measurement simulates the vehicle starting on the route with a slope of +10% (uphill). The vehicle was positioned facing uphill, and afterward the measuring equipment was applied and the vehicle was started. It gradually accelerated until it drove the entire route of the measuring section. After that, the vehicle was stopped safe and the data recording was finished. All 20 measurements were performed in the same way. The uphill measurement visualisation is shown in Figure 4.

**Figure 5:**
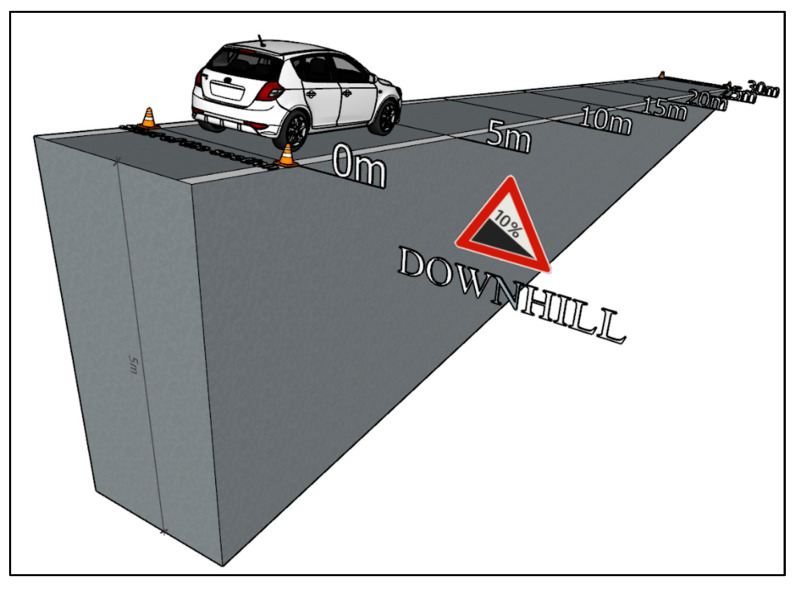
Measurement Visualization—Downhill [author via Sketchup].

In relation to this type of measurement, the vehicle drove a route with an elevation of 5 m. As the entire measuring route was given to 50 m, the resulting value of the road elevation is 10%. The values of superelevation were determined via the map client of the geographic information system ZBGIS [44]. When measuring the exhaust emissions production during a vehicle starting downhill, the same route was used as for starting uphill. The only change was the position of the vehicle, which was facing downhill. Likewise, this type of measurement was repeated 20 times. The downhill measurement visualization is shown in Figure 5.

## 3. Driving Tests Assessment

**Figure 6:**
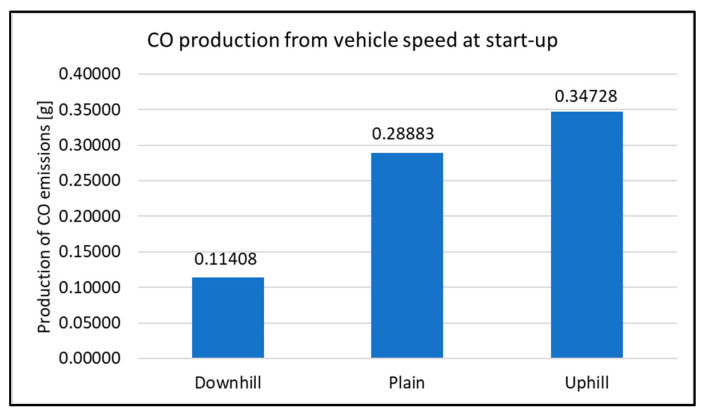
CO production at start-up.

**Figure 7:**
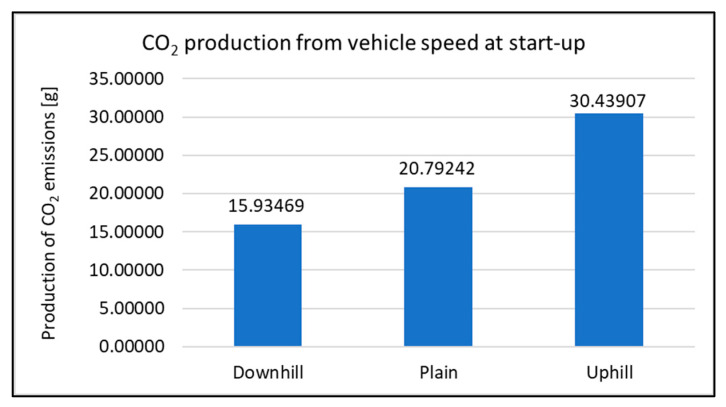
CO_2_ production at start-up.

**Figure 8:**
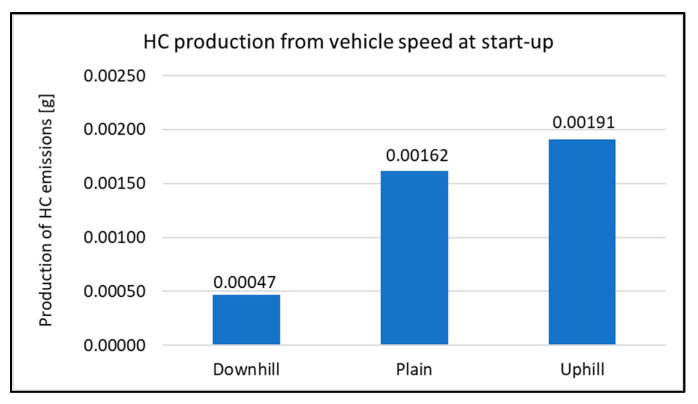
HC production at start-up.

**Figure 9:**
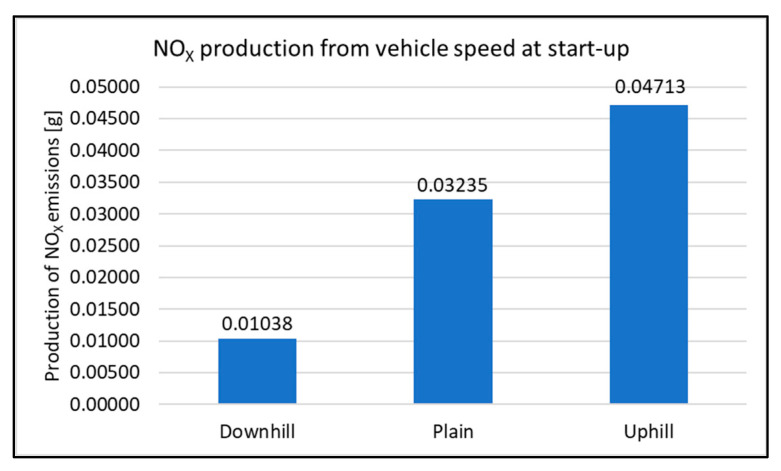
NO_X_ production at start-up.

When assessing the driving tests, the exhaust emissions production during the vehicle starting is considered. For this study, there were 20 measurements performed for each type of road. The primary results of the emissions production are shown in Figure 6, Figure 7, Figure 8 and Figure 9. All other images were captured and created by the author during testing.

It can be concluded for the CO emissions production that it was the lowest when starting “downhill”. Here, the total amount of the emissions production was 0.114 g. This was produced by a vehicle that drove 30 m. When driving uphill, the value of the emissions production was 0.347 g. To express the change as a percentage, it is almost a 200% increase in emissions production. It is necessary to highlight that the increase was caused only by a change in the road gradient. If other factors were considered (wind, higher speed and so on), the difference in emissions production would be even larger.

Concerning the CO_2_ emissions production, the changes are not so considerable. Despite that, it is possible to see the increase in emissions production in contrast to driving uphill. To say it optimally, it can be compared also to driving at ground level (plain). Relating this type of measurement, the increase in emissions is almost 47%. Concerning the overall assessment, the lowest amount of emissions was produced by the vehicle driving downhill.

There was no significant change in HC emissions compared to the other types of tests. During the measurement, the highest HC production was detected when the vehicle started uphill. This is almost a 320% increase compared to starting the vehicle downhill. When compared to the start-up of the vehicle at the ground level (plane), it is possible to observe an increase in the total production of emissions. The minimum difference is observed when starting on the plane and uphill. The percentage increase is at the level of 18%. From an overall perspective, it is possible to evaluate the vehicle’s downhill run as the best in terms of emissions production.

**Figure 10:**
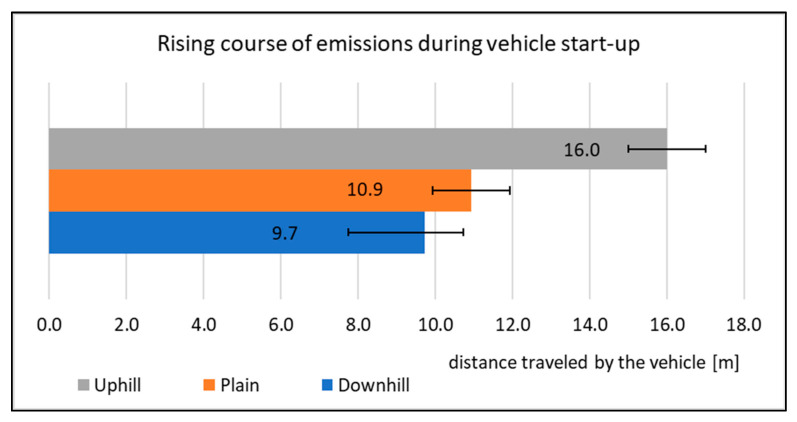
Rising course of emissions during vehicle start-up.

Based on the measurements in terms of the assessment of the driving tests, it can be concluded that a vehicle represents the lowest pressure on the environment when driving downhill. It is also proved by Figure 10, which displays the route on which the vehicle emissions have a rising course.

**Figure 11:**
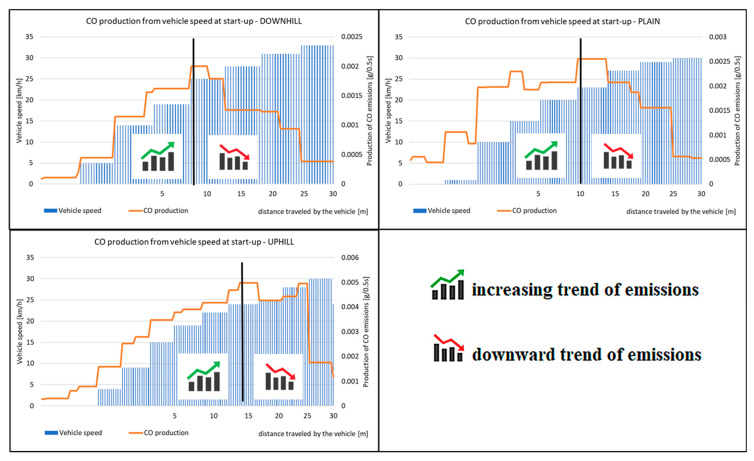
Comparison of the course of emissions for one start-up of the vehicle.

Figure 10 shows the average values of the distances driven by the vehicle during the driving tests in which the emissions have an increasing trend. As the resulting values represent the average values of the distances determined, data on the range of variation of the measured data were inserted. This range gives an interval in which the values of the distances vary during the measurement. The vehicle emissions had an increasing trend during these distances. The detailed description is shown in Figure 11, which displays the exhaust emissions production depending on vehicle speed.

**Figure 12:**
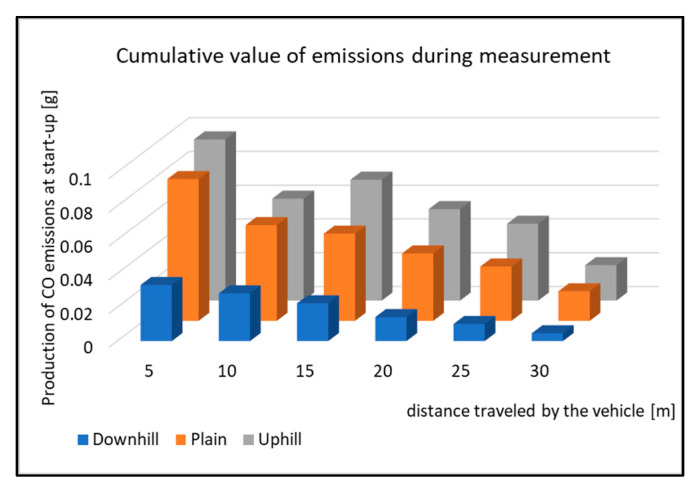
Cumulative value of emissions CO during measurement.

The rising course of the emissions during the measurements is shown in Figure 11. The values of the distance driven correspond to the interval, which is shown in Figure 10. Furthermore, it is possible to see the course of the emissions production during the start-up itself. When starting downhill, the emissions rise to 9.5 m from the start. When starting on level ground, the difference is not very visible. However, the measurement results show that the difference of the distance driven, at which the emissions are rising, is 1.1 m from the start, as shown in Figure 10. Based on a graphic display of a single start-up, Figure 11 supports this conclusion. When starting uphill, the longest driven route was observed and the emissions were rising. The average value of the distance driven is 16.00 m. To support this claim, Figure 11 displays the course of the emissions production. The comparison of CO emissions production for each driving test is shown in Figure 12.

**Figure 13:**
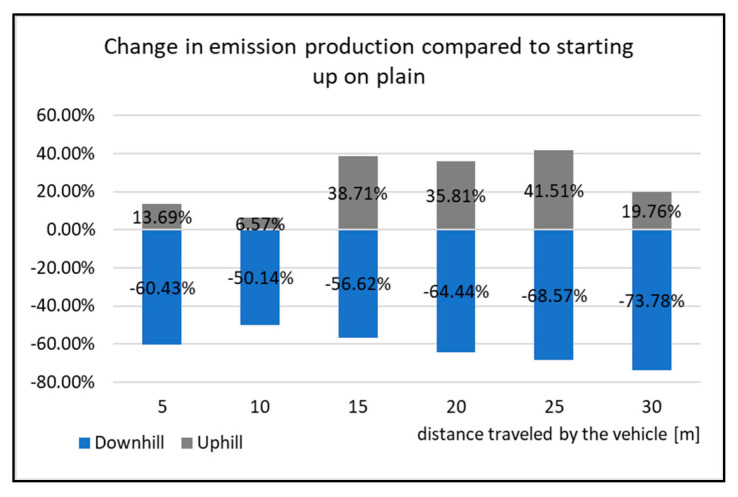
Change in emissions production compared to starting up on plain.

Figure 12 displays the cumulative values of CO emissions during the start-up tests. All the types of driving tests have the same courses of emissions, which means that the vehicle has the highest emissions production right after starting. The emissions production is then reduced, but the vehicle speed increases. This is done by shifting the gear to a higher one. As the vehicle is equipped with an automatic gear, the moment of shifting was not affected by the driver during the tests. However, it is obvious from the Figure that the emissions production is not the same when testing. The emissions production is highest while the vehicle is starting uphill. The percentage change in the emissions production during the start-up tests is shown in Figure 13.

The graph in Figure 13 displays the percentage difference of change in the emissions production compared to the vehicle starting on level ground (plain). Starting uphill had the highest change from the distance of 15 m. From this distance, the emissions production increased by 30% compared to the driving tests on level ground. The opposite occurs when starting downhill. Here, CO emissions are decreased on average by 62% compared to starting on level ground (plain). Due to this fact, it can be said that the vehicle starting downhill is the least burdensome for the environment with regards to emissions production.

**Figure 14:**
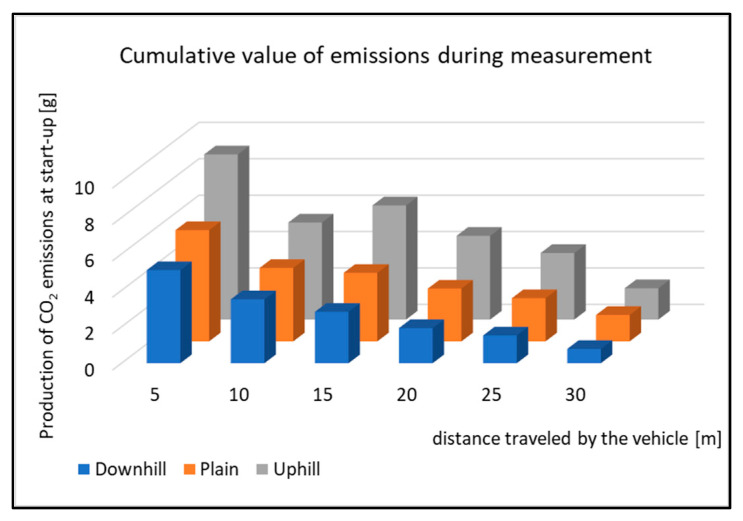
Cumulative value of emissions CO_2_ during measurement.

Figure 14 displays the cumulative values of CO_2_ emissions during the start-up tests. All the types of driving tests have very similar emissions courses, which means that the vehicle has the highest emissions production right after starting. The production is then reduced, but the vehicle speed increases. However, it is obvious from the Figure that the emissions production is not the same as when testing. The emissions production is highest while the vehicle is starting uphill. The percentage change in the emissions production during the start-up tests is shown in Figure 13.

**Figure 15:**
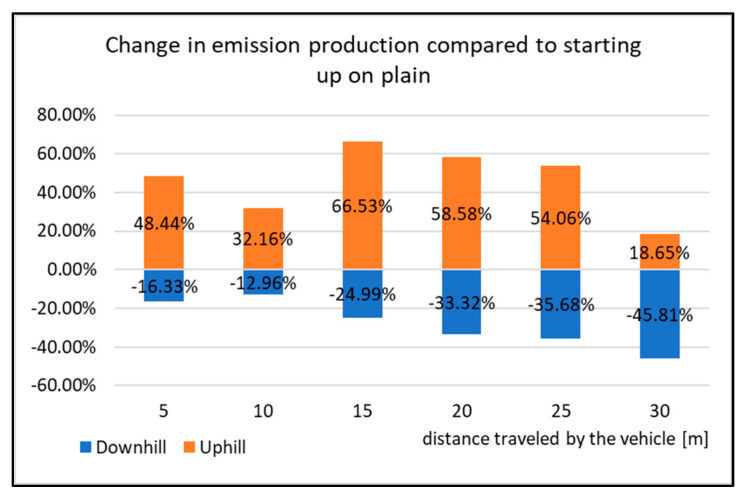
Change in emissions production compared to starting up on plain.

The graph in Figure 15 displays the percentage difference of change in the emissions production compared to the vehicle starting on level ground. When starting uphill, the highest change was from the distance of 15 m. From this distance, the emissions production increased by almost 59% compared to the start-up tests on level ground. However, when driving 30 m, the emissions production is reduced and its value increased on average by 18%. The opposite occurs when starting downhill. Here, CO emissions decrease on average by 28% compared to starting on level ground. Due to this fact, it can be said that the vehicle starting downhill is the least burdensome for the environment with regards to emissions production.

**Figure 16:**
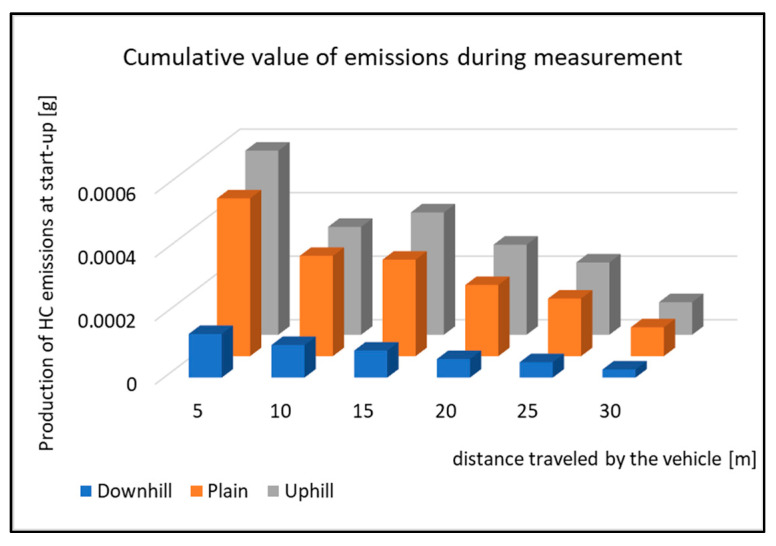
Cumulative value of HC emissions during measurement.

Figure 16 shows the production of HC emissions during the driving tests. Similar to the previous graphs, a similar course of emissions is observed. The production of HC emissions reaches the lowest values when starting downhill. However, the difference between starting the vehicle on the plane and uphill is minimal. The total value in production was already shown in Figure 8. However, in this case, it was the total emissions production for the entire route. Yet, the graph shows that the production of HC during the start uphill is the highest during the entire measurement period.

**Figure 17:**
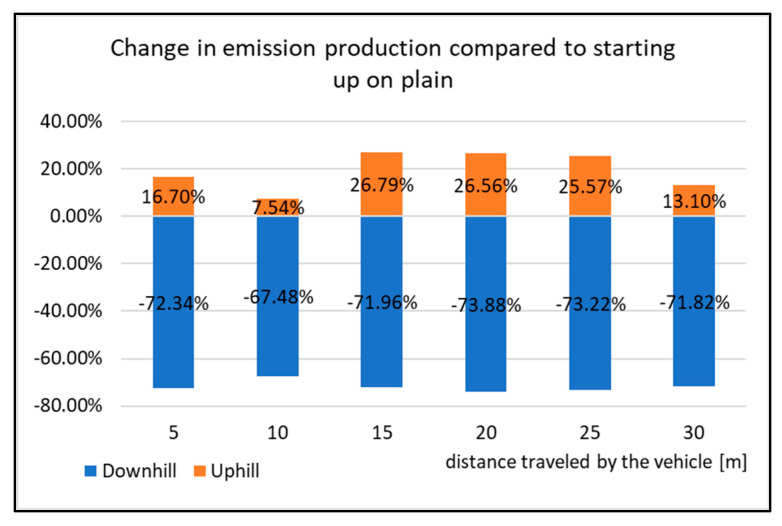
Change in emission production compared to starting up on a plain.

From the point of view of comparing the production of emissions, it is possible to observe the percentage change (Figure 17). The difference in emissions during start-up is the highest when the vehicle is starting downhill. The difference when compared to starting on the ground (plain) represents a decrease of almost 72%, which indicates that the starting of the vehicle down the slope turned out to be the most ecological again. Compared to other evaluated curves, even in Figure 17, we do not observe any significant change in HC production, even when the vehicle starts uphill. The increase in emissions when starting uphill is not extreme. In percentage terms, it represents a value of 19% compared to the vehicle’s start-up in the plain test.

**Figure 18:**
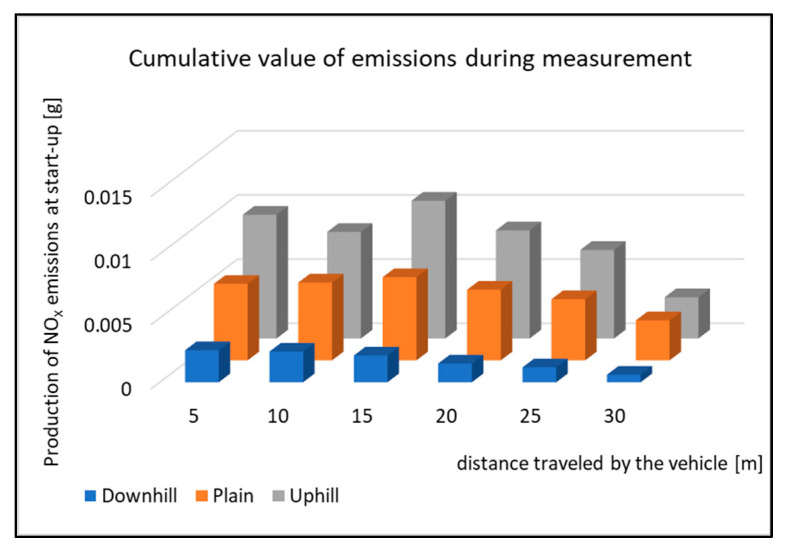
Cumulative value of Nox emissions during measurement.

**Figure 19:**
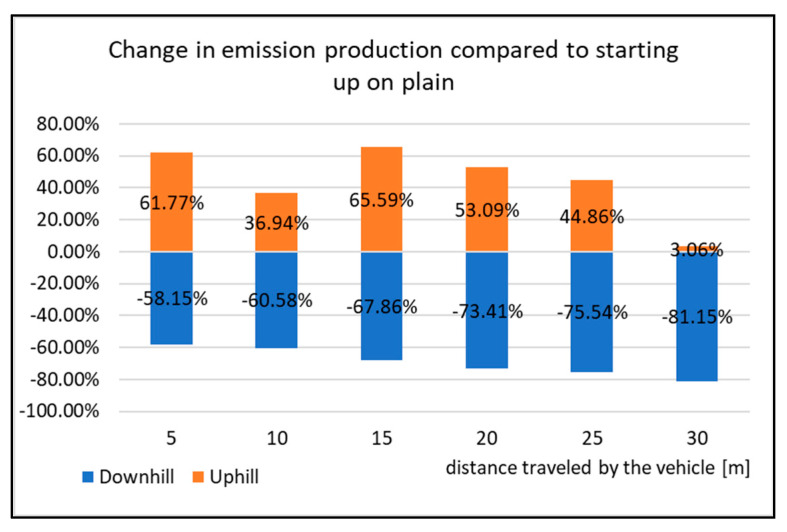
Change in emission production compared to starting up on plain.

Figure 18 shows the cumulative values of Nox emissions production during the start-up tests. All the types of driving tests have very similar emissions courses, which means that the vehicle has the highest emissions production right after starting. The production is then reduced, but the vehicle speed increases. However, it is obvious from the Figure that the emissions production is not the same as when testing. The emissions production is highest while the vehicle is starting uphill. The percentage change in emissions production during the start-up tests can be seen in Figure 19.

The graph in Figure 19 displays the percentage difference of change in the emissions production compared to the vehicle starting on level ground. When starting uphill, the highest change was from the distance of 15 m. From this distance, the emissions production increased by almost 54% compared to the start-up tests on level ground. However, when driving 30 m, the emissions production was reduced and its value increased only by 3%. The opposite occurred when starting downhill. Here, the Nox emissions decreased on average by 69% compared to starting on level ground. Due to this fact, it can be said that the vehicle starting downhill is the least burdensome for the environment with regards to emissions production.

## 4. Discussion

Nowadays, many vehicle owners are very much interested in reducing fuel consumption. The study results of Levin et al. [45] show that if drivers do not consider the gradient, they can choose a route that really increases vehicle energy consumption and, thus, the emissions production.

Studies of Costagliol et al. [46] focused on the impact of the road gradient on real emissions production while driving. Two vehicles were tested. The resulting analysis showed a significant decrease in emissions production when shifting from a positive (uphill) to a negative (downhill) slope of a road. Both vehicles tested showed similar performance in relation to emissions production. When driving with a 5% gradient, the emissions production increased by almost 100% and the fuel consumption increased proportionally. The change was seen when compared with driving on level ground. Our research has a similar change, and the change in emissions production was increased by 50% compared to driving on level ground. Conversely, the negative gradient of −4% resulted in an almost 30% decrease of CO_2_ regarding the level route. Concerning NOx emissions, the results showed very large differences in emissions production while driving uphill, on level ground and downhill.

Other studies pay attention to emissions reduction directly at the junctions [47,48,49]. The research results show that emissions reduction is not simply the equivalent of reducing the number of stops because the delays and stopping are strongly correlated in urban traffic. Based on studies of traffic flows, the delay is increased when the number of stops is reduced. The research results of Li et al. [50] show that reducing the amount of stopping can lead to CO reduction at the expense of an increase in CO_2_ and HC, whilst NOx is slightly affected, and when the amount of stopping is considerable, all four pollutants increase.

Some research [51] uses models to determine environmental contributions from transport management. Results show that better transport management in urban areas can save up to 5% of fuel, whereas higher speed on inter-city roads can save up to 2–3%, and better route guidance can save up to 3%. This indicates that better transport management can significantly reduce pollution. Concerning the road gradient, Pierson et al. [52] found that CO and NOx emissions were 2 times higher when driving uphill with a slope of approximately 4%. Kean et al. [53] discovered in their study that the increase in NOx emissions depending on vehicle speed is not so high as compared to CO emissions. In this case, it can be concluded that there is a change in emissions production that depends on the fuel consumption and average speed.

Other research results show that traffic signs at junctions create approximately 50% more emissions than roundabouts. There was even higher HC emissions production observed during congestion. The comparison was conducted with roundabouts [54]. Another similar study by Varhelyi shows that substitution of a signalizing junction with a roundabout leads to an average reduction of CO emissions by 29%, NOx emissions by 21%, and fuel by 28% per one vehicle. Mandavilli et al. [55] in their research found that a roundabout acts better than an existing junction control with stop signs in the reduction of vehicle emissions.

The research results really prove that the emissions of combustion engine vehicles depend on road slope. As in the studies [56,57], our study also proves that emissions are several times more than when driving uphill. In comparing the results of the driving test, there are changes in emissions production observed. These changes expressed in percentages are shown in Table 4.

Table 4 shows the percentage increase in emissions production compared to starting downhill. Regarding percentage changes in emissions production, it can be concluded that the largest change was in NOx emissions. They increased by as much as 354% in comparison with starting downhill. The minor change was in CO_2_ (91%); however, in this case, it is also a relatively large increase in comparison with starting downhill. The research results prove that the vehicle starting downhill is the most environmentally friendly with regards to emissions production. During this type of testing, the vehicle produced the lowest amount of emissions of all the values monitored. When monitoring the route on which the exhaust emissions have a rising character, certain changes can be observed. While starting downhill, the emissions are rising at the distance of 9.7 m. In contrast, while starting uphill, the emissions are rising even to the distance of 16 m from the start. It follows from the research that the emissions while starting uphill have an increasing trend on average by 6.3 m longer than while starting downhill.

## 5. Conclusions

In general, air quality is poor. The annual increase in passenger cars deteriorates this situation. The role of this study is to monitor emissions production during vehicle start-up. There were three types of measurements selected for the study’s objective. These include the vehicle starting downhill, on level ground (plain) and uphill. The start-up tests were repeated. The study results definitely show that the lowest amount of emissions is produced by the vehicle that is starting downhill. Overall, it has the lowest emissions production among all the pollutants monitored (CO, CO_2_, HC and NOx).

The largest difference in emissions production was seen in HC emissions. Here, there was a 71% decrease compared to starting on level ground. The lowest decrease in emissions while starting on level ground was seen in CO_2_ production. The difference in the decrease was about 28%. Although such a decrease is considered the lowest, it is a relatively large decrease in terms of emissions production. It is necessary to bear in mind that the study results can be affected by several indicators. The main ones include a route slope and a vehicle measured. Naturally, the driver’s style plays a role too. Despite all of the factors, it can be said that starting downhill is the least burdensome for the environment. In this way, during driving tests, the vehicle produced the lowest amount of emissions.

A significant parameter influencing the mass amount of produced emissions is the consumed amount of fuel mixture (air + fuel). It was at the level of 2000 g when driving on level ground (plain), 2600 g when driving uphill and 1300 g when driving downhill. The volumetric composition of emissions also affects the resulting value of their mass flow. For example, the maximum NO_X_ values of 200–250 ppm are reached when driving uphill, while they are 80–110 ppm when driving downhill. These volume concentrations obviously depend on the action parameters of the engine control unit. In this type of fuel mixture preparation control, especially from the length of the injection valve opening, the moment of fuel injection, and the ignition advance of the fuel mixture.

The research results contribute to the field of planning and designing transportation in cities. The Slovak Republic is specific for its rugged terrain. Some cities are built on hilly terrain. It follows that communications will also be conducted on hilly terrain. To ensure traffic service, it is necessary to build intersections. It is at these intersections that vehicles start uphill and downhill. This is one of the reasons for carrying out the research. The results can be further reproduced and used as background studies in the planning and construction of new intersections and places where there is a high probability of vehicles starting up. Based on the results, when designing a road network, junctions should be considered especially. After taking account of the results, it is possible to design a communication on which the starting section will be directed downhill. According to the research results, vehicles starting on this type of route produce much less emissions than when starting uphill or on level ground. The study represents a contribution to software simulations management in which the software calculates the amount of exhaust emissions produced. The results can be further presented when determining the principles of eco-driving, as well as when programming autonomous vehicles and vehicles with GPS-assisted cruise control.

## Figures and Tables

**Table 1:** Technical data of the measured vehicle [author].

Technical Parameters of the Measured Vehicle	
Brand	KIA
Trade name	Ceed
Engine code	G4FC
Number of cylinders	4
Cylinder displacement	1591 cm^3^
Highest engine power	9000 kW
Speed at max. moment	6200 min^−1^
Highest design speed	187 km·h^−1^
Millage (odometer)	150,000 km
Three-way catalytic converter	yes
Production year	2005
Fuel type	Petrol
Length	4235 mm
Width	1790 mm
Height	1480 mm
Operating weight	1191 kg
Maximum permissible total weight	1730 kg

**Table 2:** Technical data MAHA MGT 5 [40].

Technical Data MAHA MGT 5					
Measured Gases	CO	CO_2_	HC	O_2_	NOx
measuring ranges	0–15.00 Vol %	0–20.00 Vol %	0–2000 ppm Vol (Hexane) 0–4000 ppm Vol (Propane)	0–25.00 Vol %	0–5000 ppm Vol
accuracy of measuring	0.06 Vol %	0.5 Vol %	12 ppm	0.1 Vol %	32–120 ppm Vol
measurement principle	infrared	infrared	infrared	electro-chemical	electro-chemical
resolution of values	0.001	0.01	0.1	0.01	1
measuring range deviation	less than ± 0.6% of the final value of the measuring range				
Flow	max. 3.5 L/min · min 1.5 L/min				
gas outlet	approx. 2.5 L/min				
condensate drain	automatically, continuously · approx. 1 L/min				
working pressure	750–1100 mbar				
pressure fluctuations	max. error 0.2% with fluctuations of 5 kPa				

**Table 3:** Technical data of the measuring route [author].

Test Type	Plain	Uphill	Downhill
total length of the route	50 m	50 m	50 m
length of the monitored section	30 m	30 m	30 m
slope of the route	0%	10%	−10%
type of surface	asphalt	asphalt	asphalt
weather	dry, wind speed 2 m·s^−1^		
air pressure	1007 hPa		
humidity	86%		

**Table 4:** Percentage changes in exhaust gas production [author].

% Change in Emissions Increase		
	Plain	Uphill
CO	153%	204%
CO_2_	30%	91%
HC	244%	311%
NO_X_	212%	354%

## Data Availability

Data available in a publicly accessible repository. The data presented in this study are openly available in [repository name e.g., FigShare] at [doi], reference number [reference number].
